# Human animal contact, land use change and zoonotic disease risk: a protocol for systematic review

**DOI:** 10.1186/s13643-025-02805-3

**Published:** 2025-03-19

**Authors:** Aliyu N. Ahmed, Kimberly M. Fornace, Takuya Iwamura, Kris A. Murray

**Affiliations:** 1 Centre on Climate Change and Planetary Health, Medical Research Council Unit the Gambia at London School of Hygiene and Tropical Medicine, Atlantic Boulevard, Fajara, The Gambia; 2 Saw Swee Hock School of Public Health and National University Health Systems, National University of Singapore, Singapore, Singapore; 3 Department F.-A. Forel for Aquatic and Environmental Sciences, Faculty of Science, University of Geneva, Geneva, Switzerland; 4 Institute of Environmental Sciences, University of Geneva, Geneva, Switzerland; 5 Department of Forest Ecosystems and Society, College of Forestry, Oregon State University, Corvallis, USA

**Keywords:** Land use change, Human animal contact, Zoonosis, Disease emergence, Spillover

## Abstract

**Background:**

Zoonotic diseases pose a significant risk to human health globally. The interrelationship between humans, animals, and the environment plays a key role in the transmission of zoonotic infections. Human-animal contact (HAC) is particularly important in this relationship, where it serves as the pivotal interaction for pathogen spillover to occur from an animal reservoir to a human. In the context of disease emergence linked to land-use change, increased HAC as a result of land changes (e.g., deforestation, agricultural expansion, habitat degradation) is frequently cited as a key mechanism. We propose to conduct a systematic literature review to map and assess the quality of current evidence linking changes in HAC to zoonotic disease emergence as a result of land-use change.

**Method:**

We developed a search protocol to be conducted in eight (8) databases: Medline, Embase, Global Health, Web of Science, Scopus, AGRIS, Africa-Wide Info, and Global Index Medicus. The review will follow standard systematic review methods and will be reported according to the Preferred Reporting Items for Systematic reviews and Meta-Analysis (PRISMA) guidelines. The search will consist of building a search strategy, database search, and a snowballing search of references from retrieved relevant articles. The search strategy will be developed for Medline (through PubMed) and EMBASE databases. The search strategy will then be applied to all eight (8) databases. Retrieved articles will be exported to EndNote 20 where duplicates will be removed and exported to Rayyan®, to screen papers using their title and abstract. Screening will be conducted by two independent reviewers and data extraction will be performed using a data extraction form. Articles retrieved will be assessed using study quality appraisal tools (OHAT-Office for Health Assessment and Technology Risk of Bias Rating Tool for Human and Animal Studies, CCS-Case Control Studies, OCCSS-Observational Cohort and Cross-Sectional Studies, and CIS-Controlled interventional studies). Data will be analysed using descriptive statistics and a meta-analysis where data permits.

**Discussion:**

The review will provide an important systematic literature aggregate of existing evidence on the role and evidence quality linking HAC to the emergence of zoonoses via land-use change. The outcome of the proposed review will produce a high-level evidence document that could inform intervention points and further research priorities.

**Registration:**

The review will be registered with PROSPERO.

**Supplementary Information:**

The online version contains supplementary material available at 10.1186/s13643-025-02805-3.

## Introduction

Risk factors for the introduction of zoonotic diseases into human populations are often linked to the interactions between people, animal hosts, and changing socio-ecological contexts (e.g., deforestation for agriculture or urban expansion) [1]. These interactions include human animal contact (HAC), which serves as the key moment in time at which a zoonotic pathogen may have an opportunity to pass from an animal host to a human. These contacts occur through several exposure pathways collectively referred to as contact moments which includes consumption, occupational, environmental, recreational, and habitat exposure.

Human-vector contact rate is a primary parameter in vector-borne disease epidemiological models [2], and estimating this rate empirically has been a key feature of vector-borne disease studies [3, 4]. Such studies have been seminal in anticipating VBD risks which depend on parameters including vector bite rate, vector population size and distribution. HAC is the equivalent parameter in mathematical spillover models and drives both direct and indirect transmission of zoonotic pathogens [5, 6]. HAC has been estimated empirically in some studies, usually for endemic diseases including rabies [7], M. Bovis and M. tuberculosis [8]; Leptospirosis [9]; Campylobacteriosis [10], and is a well characterized contributor to the burden of these diseases in some cases [11–13]. However, in the broader context of zoonotic disease emergence, the linkage between changing HAC patterns and zoonotic disease spillover/emergence via environmental change is less clear.

Critical gaps including data on specific types of contact events, their frequency, and spatio-temporal patterns have been noted [14]. Nevertheless, there is a prevailing assumption at high policy and research governance levels (e.g., WHO, IPBES, UNEP) that increases in HAC as a result of environmental change is driving an apparent increase in zoonotic disease emergence risk in humans [15–19]. The evidence base around changing HAC patterns and disease emergence is therefore critical to clarify, particularly given that the majority of emerging as well as endemic human infectious diseases, including some that have gone onto become recent pandemics, have their origins in animal hosts [20]. A better understanding of HAC and its drivers in the context of zoonotic diseases is therefore at the crux of future disease prevention and control [21].

Existing systematic reviews have often focused narrowly on specific types of HAC or particular zoonotic diseases, limiting their ability to draw a broader conclusion on zoonotic disease risk. For instance, systematic reviews on human-livestock interactions have primarily concentrated on agricultural settings, where close contact between humans and livestock facilitates the transmission of zoonotic diseases like brucellosis and avian influenza [22, 23]. These reviews provide valuable insights into specific transmission dynamics but often neglect interactions with wildlife or companion animals, which are equally important for understanding zoonotic spillover more broadly [24, 25]. A review by Grace et al. [26] on the risks associated with human-livestock contact in Sub-Saharan Africa (SSA) highlighted the importance of livestock in zoonotic disease transmission but did not consider how land use changes or interactions with wildlife might influence these risks.

Reviews focusing on human-pet contact, such as those by Overgaauw and van Knapen [27] and Stull et al. [28], tend to emphasise on zoonotic risks within urban settings and specific demographic groups, like children or the elderly. These reviews are crucial for understanding the transmission of diseases such as toxoplasmosis and leptospirosis from pets to humans but often fail to account for how urban expansion into wildlife habitats might alter this transmission system [29, 30].

Furthermore, reviews centered on specific zoonotic diseases, for instance brucellosis or avian influenza, provide detailed analyses of pathogen transmission but lack a broader ecological and epidemiological context [31, 32]. Also, systematic reviews on avian influenza, such as those by Van Kerkhove et al. [33] and Liang et al. [34], have extensively documented the disease's transmission in poultry farms but have not integrated these findings with broader environmental changes or human-wildlife interactions, limiting their ability to generalize across different contexts.

Another limitation of the existing systematic reviews is the geographic and socio-economic bias inherent in much of the studies. Many reviews synthesize studies conducted predominantly in high-income countries or regions with well-established research infrastructures, leading to an underrepresentation of low- and middle-income countries (LMICs) [26, 35]. This is problematic because LMICs also experience rapid land use changes, such as deforestation and agricultural expansion, that can significantly influence zoonotic disease risks [36, 37]. Systematic reviews focusing on zoonotic diseases in Europe or North America, such as those by Johnson et al. [38] and Jones et al. [39], may overlook critical factors relevant to zoonotic disease emergence in regions like Africa, Asia, or Latin America.

In addition, there is a notable lack of standardization in defining and measuring key variables such as HAC, land use change, and zoonotic disease incidence across systematic reviews [39, 40]. This inconsistency complicates efforts to compare findings across studies or conduct meta-analyses that could yield more robust conclusions [41]. For instance, different reviews may use varying definitions of HAC, with some focusing on direct physical contact and others including indirect contact through vectors or the environment [42, 43]. Similarly, the categorization of land use changes is often inconsistent, with some reviews broadly categorizing changes as "urbanization" or "agricultural expansion" without considering specific types of land use changes that may have different implications for zoonotic disease risk [44, 45]. Socio-economic and cultural factors, which are crucial for understanding how different populations interact with animals and how these interactions might change due to land use changes, are often overlooked in systematic reviews [46, 47]. For example, traditional hunting practices, the use of animals in cultural rituals, or reliance on livestock for subsistence are practices that can influence the risk of zoonotic disease transmission, but these socio-cultural dimensions are often not adequately addressed [48, 49]. The review by Grace et al. [26] on zoonotic diseases in developing countries emphasized the importance of socio-economic factors in disease transmission but noted the lack of research integrating these factors into broader themes.

Also, the interdisciplinary approaches necessary to comprehensively understand zoonotic disease risk are often absent from systematic reviews [37, 39]. Most reviews are conducted within specific disciplinary silos, either focusing on ecological and biological mechanisms or socio-economic factors, without integrating these perspectives [50, 51]. For instance, the review by Estrada-Peña et al. [36] on tick-borne diseases highlighted the importance of ecological changes but did not adequately consider human behavioral factors that might influence disease transmission.

Furthermore, the relationship between HAC, land use change, and zoonotic disease transmission has not been systematically assessed on a global scale. Existing reviews on similar topics are more limited in their scope, with a focus on single species e.g., human–livestock contact [52], human-pet contact [53]; defined populations (e.g., brucellosis in humans and animals in Kenya by [54]; or specific zoonotic diseases e.g., Rift Valley Fever [55]. Here, we will build on these studies by conducting a global systematic review from eight (8) databases focusing on all HAC types/moments, which are non-disease and non-population specific, and with no restriction on year of publication. The review will also function to provide extensive scope and overview of the quality of the available evidence and capture and analyze any discrepancies between different studies. This will specifically focus on investigating the evidence of increasing HAC due to land use change, identifying associated anthropogenic activities, animal species linked to increased HAC occurrences, and investigating the presence of empirical data verifying these phenomena.

Overall, while existing systematic reviews provide valuable insights into HAC, land use change, and zoonotic disease risk, they are characterized by significant limitations, including narrow focuses on specific interactions or diseases, geographic and socio-economic biases, methodological inconsistencies, and a lack of interdisciplinary approaches. Addressing these gaps is essential for developing a more integrated understanding of how land use changes and human-animal interactions contribute to zoonotic disease emergence and transmission on a global scale. This review aims to fill these gaps by offering a systematic and interdisciplinary synthesis of existing evidence, drawing on a wide range of studies to provide a broad assessment of the factors driving zoonotic disease risk.

To comprehensively address this research gap, we developed a protocol to guide the systematic review process focusing on human-animal contact, agricultural land use change, and the associated risk of zoonotic diseases. This protocol outlines the research questions to be explored, describes the systematic literature search process, establishes criteria for study selection, defines methods for assessing study quality, outlines data extraction procedures, and the approach for analyzing data obtained from relevant studies.

## Methods

### Review question

In defining the research question, the PEO (Population, Exposures, Outcome) framework (Table 1) [56] was adopted and further refined using the FINER (Feasible, Interesting, Novel, Ethical, and Relevant) criteria for constructing a research question. Following a preliminary search, important knowledge gaps were identified, and the final research questions emerged as follows:

**Table 1:** PEO framework

Population	Human Population
Exposure	Land use change: deforestation, urban development, habitat loss, road building.
Outcome(s)	a.) Human animal contact: Types, frequency, pattern, and moments b.) Contact associated Zoonotic diseases: Viral diseases, Bacterial diseases, Fungal diseases, Parasitic diseases

i) Is there evidence that HAC (including proxies: reservoir/animal host sightings and density) increases due to land use change?
ii) What are the anthropogenic activities associated with increased HAC?
iii) What are the animal species associated with increased HAC?

The PEO framework will play a crucial role in organizing, focusing, and executing the review, ensuring it remains tightly aligned with the research questions by clearly defining the Population (human populations affected by land use changes), Exposure (land use changes like deforestation, urban development, habitat loss, and road building), and Outcomes (human-animal contact and related zoonotic diseases). This structured approach will guide the literature search, refine data extraction, and align findings with the research questions, enhancing the review's quality and clarity. It will also help ensure the data collected is relevant and comparable, making the review a valuable tool for understanding the complex interactions between land use change, human-animal contact, and zoonotic disease risk. Additionally, the PEO framework will support meta-analysis and narrative synthesis, providing a consistent basis for comparing studies and highlighting patterns or gaps in the literature.

### Review objectives

The objective of the protocol is to establish a clear procedure for gathering information to explore the knowledge gaps and answer the research questions identified for the systematic review of the existing evidence on human animal contact, agricultural land use change and zoonotic disease risk. This includes the research questions that will be answered by the systematic review, a description of the systematic literature search process, criteria for study inclusion and exclusion, study quality ratings, data extraction, and data analysis from eligible studies.

### Evidence gathering and study selection

To ensure the quality of the protocol, the review will be designed according to standard methods [57] and reported based on the Preferred Items for Systematic review and Meta-Analysis protocol (PRISMA) 2020 checklist.

### Evidence gathering

#### Search approach (Fig. 1)

**Fig. 1:**
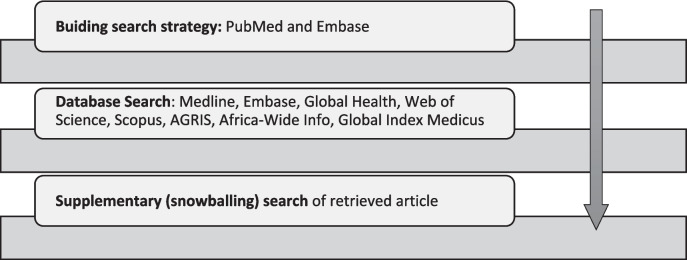
Search strategy

Stage 1 – Building search strategy in Medline through PubMed and Embase. Initial preliminary search terms will be derived from search blocks; human animal Contact, zoonosis, disease risk, and land use change/ agricultural land use change. Here, search terms will be built to each search block from analysis of article keywords, titles, abstracts, indexed terms, and subject headings.

Stage 2 – Search in all databases will be conducted in the second stage of evidence collation. Here, eight (8) databases will be searched using search terms derived from the preliminary search and tailored to specific databases’ functionality. Databases will include Medline, Embase, Global Health, Web of Science, Scopus, AGRIS, Africa-Wide Info, and Global Index Medicus.

Stage 3 – Supplementary (snowballing) search will be conducted on references of those papers that fulfil the eligibility criteria to identify any additional relevant references. This will be subjected to the same screening and selection process as other retrieved articles.

### Building search terms

key search terms will be selected to capture the broad scope of HAC, land use change, and zoonotic disease risk.

To guarantee a robust and comprehensive search strategy, various operators based on database features will be used to refine and retrieve relevant literature effectively. Boolean operators such as "AND" will be employed to combine key terms, ensuring that all specified criteria are met in the results, while "OR" will be used to include synonyms or related terms to broaden the search. To exclude irrelevant or unwanted terms, "NOT" will be applied. Proximity operators like "ADJ3" will be used to find terms within three words of each other, allowing for flexibility in how concepts are presented in the text. Additionally, wildcard operators, such as the asterisk (*) for multiple characters and the question mark (?) for single characters, will help capture variations of search terms, enhancing the breadth of the search. Truncation, using symbols like "$," will allow for the inclusion of different word endings or forms. Exact phrases will be searched using quotation marks to ensure that specific terms are found in the exact order required. Parentheses will be used to group terms and operators, controlling the order of operations within complex search queries. Lastly, field-specific operators will refine searches to specific parts of a record, such as titles, abstracts, ensuring that the most relevant literature is captured efficiently.

These terms include variations and combinations of keywords including:

*Human-Animal Contact:* ("human-animal interaction" OR "human-wildlife contact" OR "human-livestock interaction" OR "human-pet interaction").

*Land Use Change:* ("land use change" OR "deforestation" OR "urbanization" OR "agricultural expansion" OR "habitat fragmentation").

*Zoonotic Disease Risk:* ("zoonosis" OR "zoonotic disease" OR "spillover" OR "emerging infectious diseases").

Modifications of terms for Specific Databases (complete search strategy for all proposed databases are captured in supplementary material I).

### Medline

*Search Terms:* Medical Subject Headings (MeSH) and free-text terms

*Example Search:*

 ("Human animal contact*" OR "Human animal interaction*" OR "Inter?spec* contact*" OR "Cross?spec* contact*") AND ("Agricultural land use change" OR "Land use change" OR "Land cover change") AND ("Zoonos*" OR "Zoonotic" OR "Spillover") AND ("infection* OR disease* adj3 (emergence OR risk OR threat OR transmission OR outbreak)")

*Boolean Operators:* "AND" for combining concepts, "OR" for including synonyms, "NOT" to exclude irrelevant terms

*Modifiers:* Filters for publication date, study design, language, and age groups

### Embase

*Search Terms:* Emtree terms and free-text keywords

*Example Search:*

 ("Human animal contact*" OR "Human animal interaction*" OR "Inter?spec* contact*" OR "Cross?spec* contact*") AND ("Agricultural land use change" OR "Land use change" OR "Land cover change") AND ("Zoonos*" OR "Zoonotic" OR "Spillover") AND ("disease* adj3 (emergence OR spread OR exposure OR vulnerability OR transmission)")

*Boolean Operators:* "AND" for combining distinct concepts, "OR" for synonyms, "NOT" to filter out unrelated topics

*Modifiers:* Filters for geographic location, publication type, language, and study phase

### Global health

*Search Terms:* Free-text terms and subject headings specific to global health contexts

*Example Search:*

 ("Human animal contact*" OR "Human animal interaction*" OR "Inter?spec* contact*" OR "Cross?spec* contact*") AND ("Agricultural land use change" OR "Land use change" OR "Land cover change") AND ("Zoonos*" OR "Zoonotic" OR "Spillover") AND ("emerging adj3 disease* OR communicable adj3 disease*")

*Boolean Operators:* "AND" for combining concepts, "OR" for including synonyms, "NEAR" to find words close together

*Modifiers:* Utilize regional filters, disease-specific categories, and global health indicators

### Web of science

*Search Terms:* Topic and keyword searches

*Example Search:*

 ("Human animal contact*" OR "Human animal interaction*" OR "Inter?spec* contact*" OR "Cross?spec* contact*") AND ("Agricultural land use change" OR "Land use change" OR "Land cover change") AND ("Zoonos*" OR "Zoonotic" OR "Spillover") AND ("disease* adj3 (emergence OR threat OR transmission OR outbreak)")

*Boolean Operators:* "AND" to link different concepts, "OR" for alternative terms, "SAME" for search terms in the same sentence

*Modifiers:* Filters for study type, discipline, citation frequency, and geographical areas

### Scopus

*Search Terms:* Article title, abstract, and keyword searches

*Example Search:*

 ("Human animal contact*" OR "Human animal interaction*" OR "Inter?spec* contact*" OR "Cross?spec* contact*") AND ("Agricultural land use change" OR "Land use change" OR "Land cover change") AND ("Zoonos*" OR "Zoonotic" OR "Spillover") AND ("emerging adj3 disease* OR disease* adj3 risk OR epidemic* OR pandemic*")

*Boolean Operators:* "AND" for connecting concepts, "OR" for synonyms, "WITHIN" for specifying proximity

*Modifiers:* Filters for subject area, document type, language, and research funding sources

### AGRIS

*Search Terms:* Agriculture-related keywords and subject headings

*Example Search:*

 ("Human animal contact*" OR "Human animal interaction*" OR "Inter?spec* contact*" OR "Cross?spec* contact*") AND ("Agricultural land use change" OR "Land use change" OR "Land cover change") AND ("Zoonos*" OR "Zoonotic" OR "Spillover") AND ("agriculture adj5 (land use* OR change*)")

*Boolean Operators:* "AND" for combining different search concepts, "OR" for synonyms, "NOT" to eliminate non-agriculture topics

*Modifiers:* Filters for agricultural focus, language, and publication type

### Africa-wide Info

*Search Terms:* Regional keywords and subject headings

*Example Search:*

 ("Human animal contact*" OR "Human animal interaction*" OR "Inter?spec* contact*" OR "Cross?spec* contact*") AND ("Agricultural land use change" OR "Land use change" OR "Land cover change") AND ("Zoonos*" OR "Zoonotic" OR "Spillover") AND ("disease* adj3 (emergence OR risk OR exposure OR transmission OR outbreak)")

*Boolean Operators:* "AND" for concept linking, "OR" for synonyms, "NEAR" for proximity searching

*Modifiers:* Regional focus filters, language, and study type

### Global index medicus

*Search Terms:* Global health and zoonotic disease-related terms

*Example Search:*

 ("Human animal contact*" OR "Human animal interaction*" OR "Inter?spec* contact*" OR "Cross?spec* contact*") AND ("Agricultural land use change" OR "Land use change" OR "Land cover change") AND ("Zoonos*" OR "Zoonotic" OR "Spillover") AND ("communicable adj3 disease* OR disease* adj3 transmission OR pandemic OR risk")

*Boolean Operators:* "AND" for concept linking, "OR" for including alternatives, "NOT" to exclude unrelated topics

*Modifiers:* Global health focus, language, and publication type filters

#### Study selection (Fig. 2)

**Fig. 2:**
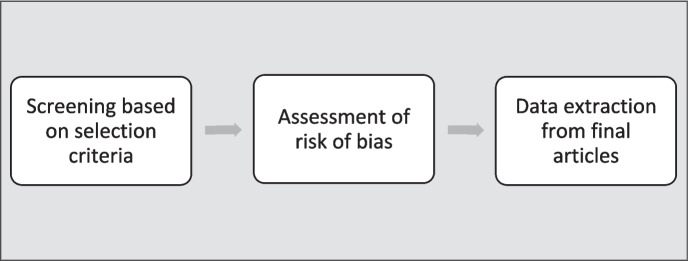
Study selection

Inclusion criteria.

- Studies on human animal contact.
- Studies linking agriculture and transmission or emergence or spillover of zoonotic diseases.
- Studies focusing on the mechanism, risk factors, and pathways of transmission of zoonotic infections from animals to humans will be considered.
- Studies on biodiversity and human animal contact.
- Included studies could be primary sources, case reports, observational studies, cross-sectional studies, cohort studies, case–control studies, and systemic review/meta-analyses.
- No restriction on geographic area.
- No restriction on the date of publication of an article.
- Searches will be limited to peer-reviewed full text articles.

Exclusion criteria

- Articles not focusing on human-animal contact.
- Studies not in English language.
- Conference papers, commentaries, opinion papers, abstracts, and editorials.
- Articles focusing on molecular studies or pathophysiology of zoonotic diseases.
- Articles not focusing on spillover or transmission of diseases from animals to humans.
- Articles not focusing on land use and zoonotic infections.

### Screening of articles

Articles will be exported to Endnote [58], where duplicates will be removed and exported to Rayyan [59], a web-based tool that aids the review screening process for articles, allowing collaborations between multiple reviewers to screen using the title and abstract of papers to ensure a critically appraised process with less bias. The screening will be conducted by two independent reviewers to ensure quality control. In the event two reviewers have a conflicting decision during the review process, a third reviewer will be consulted to resolve the discrepancies. Screening will be done in two phases, in the first phase, the reviewers will carry out “Title and abstract screening”. The references of the selected articles will be manually searched for relevant articles that will be subject to the same screening phase. Full-text articles will then be subjected to the second stage, “Full-text screening” using the same screening criteria for the previous selected articles.

### Assessment of risk of bias

Articles included in the review will be subjected to quality assessment using study quality assessment methodology tools by two independent reviewers. Four (4) Risk of Bias (RoB) assessment tools will be applied in stages based on study design including: OHAT (Office for Health Assessment and Technology Risk of Bias Rating Tool for Human and Animal Studies) [60], CCS (Case Control Studies) [61], OCCSS (Observational Cohort and Cross- Sectional Studies), and **CIS** (Controlled interventional studies) [62]. These tools will be applied in two stages, 1.) during the assessment of individual articles and 2.) when assessing article results during extraction of data. This method follows the recommendation of the Cochrane collaboration [63]. The OHAT tool can be applied to six study designs including EA: Experimental Animal, HCT: Human Controlled Trial, Co: Cohort, CaCo: Case–Control, CrSe: Cross-sectional, and CaS: Case Series/Case report studies. It has eleven (11) risk of bias questions which are rated by selecting among 4 possible answers. These include (+ +) Definitely Low risk of bias, ( +) Probably Low risk of bias, (-) Probably High risk of bias, (–) Definitely High risk of bias. The system for answering each risk-of-bias question requires reviewers to choose between low and high risk of bias options. The questions will be applied based on the type of study article assessed. The OCCSS, CCS, and CIS tools have 14, 12 & 14 questions respectively to assess the overall quality of studies using various standard established criteria such as research questions, objectives, sample justification, exposures/outcomes validity and reliability etc. These questions will be answered with yes or no responses by reviewers and the overall quality rating will be denoted Good, Fair or Poor.

### Data extraction (Table 2)

Data will be extracted by two independent reviewers using Google Forms designed to retrieve relevant information to answer the review questions.

**Table 2:** Data extraction forms

Data extraction form	
Publication details	Title Author Year Country Region Publication type
Focus of research	Research objective Phenomena/outcome(s) of interest Disease(s) Country and continent
Methodology	Type of study Study designs included Types of evidence included
Findings	Human-animal contact Drivers for zoonotic spillover Exposure factor Host reservoir Vector Intermediate host
Additional information	

### Proposed data analysis

#### Descriptive analysis

The data analysis will be conducted to comprehensively address the research questions. Initially, the focus will be on descriptive statistics to provide a detailed characterization and summary of the data. This phase involves the systematic organization of the collected data to align with the PEO framework—Population, Exposure, Outcome. Each study's key variables, including the types and frequencies of human-animal contact (HAC), land use changes, and reported zoonotic diseases, will be meticulously cataloged. Descriptive statistics, such as measures of central tendency (mean, median) and dispersion (range, standard deviation), will be computed to offer an overview of the distribution of these variables across studies. To explore the relationships between categorical variables, such as land use changes and their association with specific zoonotic diseases, frequency distributions and cross-tabulations will be utilized. Visualization techniques will further elucidate the data, employing various graphical representations including bar charts, histograms, pie charts, and geographical maps. These visualizations will aid in depicting the spatial distribution of land use changes and HAC incidents, and in examining trends or correlations over time through line charts and scatter plots.

#### Meta-analysis

If data demonstrate sufficient homogeneity, a meta-analysis will be undertaken to aggregate and synthesize findings across studies. The decision to proceed with a meta-analysis will hinge on several critical factors. First, there must be a high degree of similarity in the populations, exposures, and outcomes of the included studies, which will be evaluated through qualitative comparisons and statistical tests for heterogeneity, such as Cochran’s Q test and the I^2^ statistic. Additionally, the feasibility of a meta-analysis depends on the availability of effect sizes or sufficient data to calculate them, reflecting the relationship between land use changes, HAC, and the risk of zoonotic disease transmission. Consistency in measurement methods across studies is also essential to ensure that results can be meaningfully synthesized. This encompasses uniformity in defining and reporting HAC, land use changes, and zoonotic diseases.

If these conditions are satisfied, the meta-analysis will involve extracting or calculating effect sizes from each study, which may include odds ratios, relative risks, or other relevant measures depending on the outcome variables. These effect sizes will be pooled using either a fixed-effect or random-effects model, based on the degree of heterogeneity observed. The selection of the model will be guided by statistical criteria and the specific characteristics of the data. An assessment of heterogeneity will be conducted through statistical tests, with subgroup analyses or meta-regression employed to explore potential sources of variability, such as geographic differences or types of land use change. Sensitivity analyses will test the robustness of the meta-analysis results, which may involve excluding studies with high risk of bias or applying alternative statistical models to evaluate result stability. Additionally, the potential for publication bias will be assessed using funnel plots and Egger’s test to ensure that the meta-analysis findings are not skewed by selective reporting. The results will be presented in both tabular and graphical formats, including forest plots to illustrate pooled effect sizes and confidence intervals.

If a meta-analysis is not feasible, a narrative synthesis will be conducted. This will be structured according to the PEO framework, focusing on the interplay between land use changes, HAC, and zoonotic diseases. The narrative synthesis will explore key patterns, identify gaps in the literature, and highlight areas for further research. This comprehensive approach, integrating descriptive and meta-analytic methods as appropriate, will ensure a thorough examination of the relationship between land use change, human-animal contact, and the risk of zoonotic diseases.

### Reporting

Findings will be reported using the guidelines of the Preferred Reporting Items for Systematic Reviews and Meta-analysis (PRISMA).

## Discussion

We propose to review peer reviewed literature that will function to provide an overview of the available evidence on the role that HAC plays in the emergence and transmission of zoonotic infections, how land use change influences these contacts, and how it is linked to zoonotic infections.

This protocol has identified several critical gaps in the current understanding of the relationship between human-animal contact (HAC), land use change, and zoonotic disease risk. By synthesizing findings from existing studies, our review aims to address these gaps and offer clear guidance for future research and policy.

Our review aims to bridge these gaps by providing a more comprehensive analysis that incorporates a wider range of animal interactions and considers the impact of land use changes across different contexts. By addressing both ecological and socio-economic factors, our findings will offer a more comprehensive understanding of how these variables influence zoonotic disease transmission. This approach will not only enhance the accuracy of risk assessments but also inform targeted interventions in diverse settings.

To guide future research, we propose a multidimensional framework that integrates ecological, biological, and socio-economic factors. This framework will facilitate the development of more robust research designs and methodological approaches, addressing the inconsistencies and biases observed in current reviews [31, 32]. Additionally, our review underscores the need for studies that explore the interplay between land use changes, HAC, and zoonotic risks in underrepresented regions, particularly in low- and middle-income countries [25, 33].

Our findings will have significant implications for policy and practice. By identifying key factors that influence zoonotic disease risk, our review will provide evidence-based recommendations for policymakers and public health practitioners. For example, understanding how land use changes impact HAC and zoonotic disease risk can guide the development of land use planning and environmental management strategies that mitigate disease transmission. Additionally, our review will highlight areas where targeted interventions are needed, such as improving animal health surveillance and enhancing community awareness about zoonotic risks.

### Methodological limitations and management

The review methodology itself has certain limitations that need to be acknowledged. One significant issue is publication bias, which can skew the results by favoring studies with positive or significant findings. To mitigate this, we will actively seek grey literature and unpublished studies to ensure a more balanced representation of the available evidence [64, 65]. This includes contacting researchers directly and exploring relevant databases and repositories for grey literature.

Additionally, we acknowledge that our research protocol has specific limitations. Grey literature will not be included, and only studies published in English will be considered. While this monolingual language restriction may introduce some bias, we anticipate that it will not be significant due to the broad scope of the review and the extensive range of databases searched, which should help to mitigate the impact of this restriction [66]. Another limitation is the variability in study quality and reporting standards, which can affect the reliability of the synthesized results. We will address this by applying rigorous inclusion criteria and quality assessment tools to evaluate the methodological rigor of the studies included in the review [67, 68]. Additionally, we will consider the potential impact of heterogeneity across studies and perform sensitivity analyses to assess the robustness of our findings [69, 70]. Importantly, it will also provide a synthesis of the sparsely documented socioeconomic and environmental variables that govern these interactions and their implications in various settings. The proposed review to the best of our knowledge will be the first to summarise existing literature on human animal contact, land use change, and zoonotic disease at a global scale, and not restricted to a specific disease or species. It will therefore provide a novel high-level synthesis from peer reviewed literature and highlight research priorities by identifying research gaps for further investigative endeavours.

## Supplementary Information

Supplementary Material 1

## Data Availability

Not applicable.
