# Supplementary material for: Human animal contact, land use change and zoonotic disease risk: a protocol for systematic review
Source: Syst Rev. 2025 Mar 19;14:65. doi: 10.1186/s13643-025-02805-3 (PMC11921583; doi:10.1186/s13643-025-02805-3)
Supplement: Supplementary file 1 — Supplementary Material 1 [file 13643_2025_2805_MOESM1_ESM.docx]

1. **Supplementary material I**

Database Search Strategy

[Ovid MEDLINE(R) ALL <1946 to January 27, 2022>]

| **Search** | **Query** |  |
| --- | --- | --- |
| 1 | Human animal contact.ab,kw,ti. | 30 |
| 2 | Human animal interaction.ab,kw,ti. | 270 |
| 3 | (Human animal contact* or human animal interact* Inter?spec* contact* or cross?spec* contact*).ab,kw,ti. | 33 |
| 4 | ((interspecies or inter-species or cross-species or cross species) adj3 (contact* or transmission* or interaction* or transfer*)).ab,kw,ti. | 4,285 |
| 5 | ((human* adj3 (animal* or wildlife or livestock or fauna)) and (contact* or transmission* or interaction* or transfer*)).ab,kw,ti. | 18,700 |
| 6 | 1 or 2 or 3 or 4 or 5 | 22,650 |
| 7 | Zoonoses/ | 18,137 |
| 8 | (zoonos* or zoonotic or spillover).ab,kw,ti. | 40,302 |
| 9 | 7 or 8 | 48,959 |
| 10 | ((infection* or disease*) adj3 (emergence or emerge or emerged or emerging or risk or risks or threat* or exposure* or vulnerab* or transmi* or spread* or outbreak* or risk)).ab,kw,ti. | 330,422 |
| 11 | (communicable diseas* or diseas* outbreak* or emerging diseas* or diseas* hotspot* or epidemic* or pandemic* or community?acquired infection*).ab,kw,ti. | 269,804 |
| 12 | 10 or 11 | 562,722 |
| 13 | Agriculture/ | 47,356 |
| 14 | "Agricultural land use change".ab,kw,ti. | 38 |
| 15 | (agricultur* or farm* or biodiversity).ab,kw,ti. | 222,630 |
| 16 | agriculture/ or agricultural irrigation/ or animal husbandry/ or crop production/ or farms/ or gardens/ | 75,380 |
| 17 | "Land use change".ab,kw,ti. | 2,730 |
| 18 | "land cover change".ab,kw,ti. | 693 |
| 19 | ((Land or lands) adj5 (use* or change*)).ab,kw,ti. | 20,863 |
| 20 | 13 or 14 or 15 or 16 or 17 or 19 | 268,737 |
| 21 | 6 and 9 and 12 and 20 | 330 |

Embase Classic+Embase <1947 to 2022 January 27>

| **#** | **Query** | **Results from 28 Jan 2022** |
| --- | --- | --- |
| 1 | human animal contact.ab,kw,ti. | 36 |
| 2 | Human animal interaction.ab,kw,ti. | 392 |
| 3 | (Human animal contact* or human animal interact* Inter?spec* contact* or cross?spec* contact*).ab,kw,ti. | 40 |
| 4 | ((interspecies or inter-species or cross-species or cross species) adj3 (contact* or transmission* or interaction* or transfer*)).ab,kw,ti. | 4,478 |
| 5 | ((human* adj3 (animal* or wildlife or livestock or fauna)) and (contact* or transmission* or interaction* or transfer*)).ab,kw,ti. | 22,126 |
| 6 | 2 or 3 or 4 or 5 | 26,327 |
| 7 | zoonosis/ | 19,786 |
| 8 | (zoonos* or zoonotic or spillover).ab,kw,ti. | 44,625 |
| 9 | 7 or 8 | 53,944 |
| 10 | ((infection* or disease*) adj3 (emergence or emerge or emerged or emerging or risk or risks or threat* or exposure* or vulnerab* or transmi* or spread* or outbreak* or risk)).ab,kw,ti. | 446,385 |
| 11 | (communicable diseas* or diseas* outbreak* or emerging diseas* or diseas* hotspot* or epidemic* or pandemic* or community?acquired infection*).ab,kw,ti. | 303,937 |
| 12 | 10 or 11 | 709,125 |
| 13 | (agricultur* or farm* or biodiversity).ab,kw,ti. | 246,567 |
| 14 | agriculture/ or agricultural irrigation/ or animal husbandry/ or crop production/ or farms/ or gardens/ | 103,587 |
| 15 | "agricultural land use change".ab,kw,ti. | 37 |
| 16 | "land-use change".ab,kw,ti. | 2,887 |
| 17 | ((Land or lands) adj5 (use* or change*)).ab,kw,ti. | 22,857 |
| 18 | "land cover change".ab,kw,ti. | 691 |
| 19 | agriculture/ | 51,830 |
| 20 | 13 or 14 or 15 or 16 or 17 | 304,626 |
| 21 | 6 and 9 and 12 and 20 | 335 |

Global Health <1910 to 2022 Week 04>

| **Search** | **Query** | **Results from 28 Jan 2022** |
| --- | --- | --- |
| 1 | human animal contact.mp. [mp=abstract, title, original title, broad terms, heading words, identifiers, cabicodes] | 16 |
| 2 | Human animal interaction.mp. [mp=abstract, title, original title, broad terms, heading words, identifiers, cabicodes] | 105 |
| 3 | (Human animal contact* or human animal interact* Inter?spec* contact* or cross?spec* contact*).mp. [mp=abstract, title, original title, broad terms, heading words, identifiers, cabicodes] | 18 |
| 4 | ((interspecies or inter-species or cross-species or cross species) adj3 (contact* or transmission* or interaction* or transfer*)).mp. [mp=abstract, title, original title, broad terms, heading words, identifiers, cabicodes] | 1,483 |
| 5 | ((human* adj3 (animal* or wildlife or livestock or fauna)) and (contact* or transmission* or interaction* or transfer*)).mp. [mp=abstract, title, original title, broad terms, heading words, identifiers, cabicodes] | 24,104 |
| 6 | 1 or 2 or 3 or 4 or 5 | 25,281 |
| 7 | zoonosis/ | 53,727 |
| 8 | (zoonos* or zoonotic or spillover).mp. [mp=abstract, title, original title, broad terms, heading words, identifiers, cabicodes] | 63,152 |
| 9 | 7 or 8 | 63,152 |
| 10 | ((infection* or disease*) adj3 (emergence or emerge or emerged or emerging or risk or risks or threat* or exposure* or vulnerab* or transmi* or spread* or outbreak* or risk)).mp. [mp=abstract, title, original title, broad terms, heading words, identifiers, cabicodes] | 235,181 |
| 11 | (communicable diseas* or diseas* outbreak* or emerging diseas* or diseas* hotspot* or epidemic* or pandemic* or community?acquired infection*).mp. [mp=abstract, title, original title, broad terms, heading words, identifiers, cabicodes] | 214,099 |
| 12 | 10 or 11 | 400,035 |
| 13 | agriculture/ | 6,475 |
| 14 | (agricultur* or farm* or biodiversity or "agricultural land use change").mp. [mp=abstract, title, original title, broad terms, heading words, identifiers, cabicodes] | 180,947 |
| 15 | agriculture/ or agricultural irrigation/ or animal husbandry/ or crop production/ or farms/ or gardens/ | 14,510 |
| 16 | "land-use change".mp. [mp=abstract, title, original title, broad terms, heading words, identifiers, cabicodes] | 795 |
| 17 | "land cover change".mp. [mp=abstract, title, original title, broad terms, heading words, identifiers, cabicodes] | 97 |
| 18 | ((Land or lands) adj5 (use* or change*)).mp. [mp=abstract, title, original title, broad terms, heading words, identifiers, cabicodes] | 8,772 |
| 19 | 13 or 14 or 15 or 16 or 17 or 18 | 188,431 |
| 20 | 6 and 9 and 12 and 19 | 831 |

Web of Science

| **Search** | **Query** | **Results** |
| --- | --- | --- |
| 1 | ALL=(Human animal interaction) | 44,606 |
| 2 | ALL=(Human animal contact) | 12,856 |
| 3 | ALL=((Human animal contact* or human animal interact* Inter?spec* contact* or cross?spec* contact*)) | 13,004 |
| 4 | ALL=(((interspecies or inter-species or cross-species or cross species) AND (contact* or transmission* or interaction* or transfer*))) | 28,652 |
| 5 | ALL=(((human* AND (animal* or wildlife or livestock or fauna)) AND (contact* or transmission* or interaction* or transfer*))) | 102,580 |
| 6 | #1 OR #2 OR #3 OR #4 OR #5 | 134,253 |
| 7 | ALL=(("zoonosis*" OR "zoonoses*" OR "zoonotic*" OR "zoonos*" OR "spillover*" OR "epidemi*" OR "pandemi*")) | 1,644,502 |
| 8 | ALL=(("disease risk*" OR "Disease exposure*" OR "Infection exposure*" OR "Disease hazard*" OR "Infection hazard*" OR "Disease threat*" OR "Infection threat*" OR "Disease vulnerab*" OR "Disease transmission*" OR "Transmission risk*" OR "Transmission of infection*" OR " risk of transmission*" OR "Infection spread*" OR "Transm* of dis*" OR “inter$species contact*” OR “inter$species interact*” OR “inter$species transmission*” OR “cross species contact*” OR “cross species interact*” OR “cross species transmission*” OR ((“Human-animal*” OR “animal-human*” OR “Human to animal*” OR “animal to human*” OR “Human and animal*” OR “animal and human*” OR “human-wildlife” OR “wildlife-human” OR “human-livestock” OR “livestock-human” OR “human-fauna” OR “fauna-human”) AND (“contact” OR “interact*” OR “transmission”))) ) | 1,246,023 |
| 9 | ALL=(("Agricultur*" OR "Agricultural land use change" OR "Agricultural land-use change" OR "Agriculture land use change" OR "Agriculture land-use change" OR "Land use change" OR "Land-use change" OR "Land?use change" OR "Land$use change" OR "Land change" OR "Land cover change" OR "Land$cover change" OR "Land?change" OR "Land use" OR "Land cover")) | 75764 |
| 10 | #6 AND #7 AND #8 AND #9 | 667 |

SCOPUS

| **Search** | **Query** | **Result** |
| --- | --- | --- |
| 1 | ALL ( human AND animal AND contact* OR human AND animal AND interact* AND inter$spec* AND contact* OR cross?spec* AND contact* ) | 9097 |
| 2 | ALL ( interspecies OR inter-species OR cross-species OR cross AND species W/3 contact* OR transmission* OR interaction* OR transfer* ) | 35398 |
| 3 | ALL ( ( ( human* W/3 ( animal* OR wildlife OR livestock OR fauna ) ) AND ( contact* OR transmission* OR interaction* OR transfer* ) ) ) | 321855 |
| 4 | #1 AND #2 AND #3 |  |
| 5 | ( ALL ( human AND animal AND contact* OR human AND animal AND interact* AND inter$spec* AND contact* OR cross?spec* AND contact* ) ) OR ( ALL ( interspecies OR inter-species OR cross-species OR cross AND species W/3 contact* OR transmission* OR interaction* OR transfer* ) ) OR ( ALL ( ( ( human* W/3 ( animal* OR wildlife OR livestock OR fauna ) ) AND ( contact* OR transmission* OR interaction* OR transfer* ) ) ) ) | 359884 |
| 6 | TITLE-ABS-KEY ( ( ( "zoonosis*" OR "zoonoses*" OR "zoonotic*" OR "zoonos*" OR "spillover*" OR "epidemi*" OR "pandemi*" ) ) ) | 1,364,534 |
| 7 | TITLE-ABS-KEY ( "disease risk*" OR "Disease exposure*" OR "Infection exposure*" OR "Disease hazard*" OR "Infection hazard*" OR "Disease threat*" OR "Infection threat*" OR "Disease vulnerab*" OR "Disease transmission*" OR "Transmission risk*" OR "Transmission of infection*" OR " risk of transmission*" OR "Infection spread*" OR "Transm* of dis*" OR "inter$species contact*" OR "inter$species interact*" OR "inter$species transmission*" OR "cross species contact*" OR "cross species interact*" OR "cross species transmission*" OR "Human-animal*" OR "animal-human*" OR "Human to animal*" OR "animal to human*" OR "Human and animal*" OR "animal and human*" OR "human-wildlife" OR "wildlife-human" OR "human-livestock" OR "livestock-human" OR "human-fauna" OR "fauna-human" AND "contact" OR "interact*" OR "transmission" ) | 177,456 |
| 8 | TITLE-ABS-KEY ( "Agricultur*" OR "Agricultural land use change" OR "Agricultural land-use change" OR "Agriculture land use change" OR "Agriculture land-use change" OR "Land use change" OR "Land-use change" OR "Land?use change" OR "Land$use change" OR "Land change" OR "Land cover change" OR "Land$cover change" OR "Land?change" OR "Land use" OR "Land cover" ) | 940,429 |
| 9 | #4 AND #5 AND #6 AND #7 |  |
| 10 | ( ( ALL ( human AND animal AND contact* OR human AND animal AND interact* AND inter$spec* AND contact* OR cross?spec* AND contact* ) ) OR ( ALL ( interspecies OR inter-species OR cross-species OR cross AND species W/3 contact* OR transmission* OR interaction* OR transfer* ) ) OR ( ALL ( ( ( human* W/3 ( animal* OR wildlife OR livestock OR fauna ) ) AND ( contact* OR transmission* OR interaction* OR transfer* ) ) ) ) ) AND ( TITLE-ABS-KEY ( ( ( "zoonosis*" OR "zoonoses*" OR "zoonotic*" OR "zoonos*" OR "spillover*" OR "epidemi*" OR "pandemi*" ) ) ) ) AND ( TITLE-ABS-KEY ( "disease risk*" OR "Disease exposure*" OR "Infection exposure*" OR "Disease hazard*" OR "Infection hazard*" OR "Disease threat*" OR "Infection threat*" OR "Disease vulnerab*" OR "Disease transmission*" OR "Transmission risk*" OR "Transmission of infection*" OR " risk of transmission*" OR "Infection spread*" OR "Transm* of dis*" OR "inter$species contact*" OR "inter$species interact*" OR "inter$species transmission*" OR "cross species contact*" OR "cross species interact*" OR "cross species transmission*" OR "Human-animal*" OR "animal-human*" OR "Human to animal*" OR "animal to human*" OR "Human and animal*" OR "animal and human*" OR "human-wildlife" OR "wildlife-human" OR "human-livestock" OR "livestock-human" OR "human-fauna" OR "fauna-human" AND "contact" OR "interact*" OR "transmission" ) ) AND ( TITLE-ABS-KEY ( "Agricultur*" OR "Agricultural land use change" OR "Agricultural land-use change" OR "Agriculture land use change" OR "Agriculture land-use change" OR "Land use change" OR "Land-use change" OR "Land?use change" OR "Land$use change" OR "Land change" OR "Land cover change" OR "Land$cover change" OR "Land?change" OR "Land use" OR "Land cover" ) ) | 907 |

Africa Wide Info

| **Search** | **Query** | **Results** |
| --- | --- | --- |
| 1 | (Human animal contact* or human animal interact* Inter?spec* contact* or cross?spec* contact*) | 9 |
| 2 | ((interspecies or inter-species or cross-species or cross species) N3 (contact* or transmission* or interaction* or transfer*)) | 520 |
| 3 | ((human* N3 (animal* or wildlife or livestock or fauna)) and (contact* or transmission* or interaction* or transfer*)) | 2,883 |
| 4 | S1 OR S2 OR S3 | 3,325 |
| 5 | "zoonosis*" OR "zoonoses*" OR "zoonotic*" OR "zoonos*" OR "spillover*" OR "epidemi*" OR "pandemi*" | 178,863 |
| 6 | (("disease risk*" OR "Disease exposure*" OR "Infection exposure*" OR "Disease hazard*" OR "Infection hazard*" OR "Disease threat*" OR "Infection threat*" OR "Disease vulnerab*" OR "Disease transmission*" OR "Transmission risk*" OR "Transmission of infection*" OR " risk of transmission*" OR "Infection spread*" OR "Transm* of dis*" OR “inter$species contact*” OR “inter$species interact*” OR “inter$species transmission*” OR “cross species contact*” OR “cross species interact*” OR “cross species transmission*” OR ((“Human-animal*” OR “animal-human*” OR “Human to animal*” OR “animal to human*” OR “Human and animal*” OR “animal and human*” OR “human-wildlife” OR “wildlife-human” OR “human-livestock” OR “livestock-human” OR “human-fauna” OR “fauna-human”) AND (“contact” OR “interact*” OR “transmission”))) | 14,435 |
| 7 | (("Agricultur*" OR "Agricultural land use change" OR "Agricultural land-use change" OR "Agriculture land use change" OR "Agriculture land-use change" OR "Land use change" OR "Land-use change" OR "Land$use change" OR "Land$use change" OR "Land change" OR "Land cover change" OR "Land$cover change" OR "Land?change" OR "Land use" OR "Land cover")) | 260,052 |
| 8 | S5 AND S6 AND S7 AND S8 | 163 |

Global Index Medicus

| **Search** | **Query** | **Results** |
| --- | --- | --- |
| 1 | (tw:(Zoonosis)) OR (tw:((zoonos* or zoonotic or spillover*))) OR (tw:(((human* AND (animal* or wildlife or livestock or fauna)) AND (contact* or transmission* or interaction* or transfer*)))) OR (tw:(((interspecies or inter-species or cross-species or cross species) AND (contact* or transmission* or interaction* or transfer*)))) | 13,977 |
| 2 | (tw:(((infection* or disease*) AND (emergence or emerge or emerged or emerging or risk or risks or threat* or exposure* or vulnerab* or transmi* or spread* or outbreak* or risk)))) OR (tw:((communicable diseas* or diseas* outbreak* or emerging diseas* or diseas* hotspot* or epidemic* or pandemic* or community?acquired infection*))) | 211,228 |
| 3 | (tw:(((Land or lands) AND (use* or change*)))) OR (tw:((agricultur* or farm* or biodiversity))) OR (tw:(agriculture/ or agricultural irrigation or animal husbandry or crop production or farms or gardens)) | 395,853 |
| 4 | ((tw:(Zoonosis)) OR (tw:((zoonos* or zoonotic or spillover*))) OR (tw:(((human* AND (animal* or wildlife or livestock or fauna)) AND (contact* or transmission* or interaction* or transfer*)))) OR (tw:(((interspecies or inter-species or cross-species or crossspecies) AND (contact* or transmission* or interaction* or transfer*)))))) AND (tw:((tw:(((infection* or disease*) AND (emergence or emerge or emerged or emerging or risk or risks or threat* or exposure* or vulnerab* or transmi* or spread* or outbreak* or risk)))) OR (tw:((communicable diseas* or diseas* outbreak* or emerging diseas* or diseas* hotspot* or epidemic* or pandemic* or community?acquired infection*))) )) AND (tw:((tw:(((Land or lands) AND (use* or change*)))) OR (tw:((agricultur* or farm* or biodiversity))) OR (tw:(agriculture/ or agricultural irrigation or animal husbandry or crop production or farms or gardens)) )) | 2,394 |

**PRISMA-P 2015 Checklist**

| **Section/topic** | **#** | **Checklist item** | **Information reported** | | **Line number(s)** |
| --- | --- | --- | --- | --- | --- |
|  |  |  | **Yes** | **No** |  |
| **ADMINISTRATIVE INFORMATION** | | | | | |
| **Title** | | | | | |
| Identification | 1a | Identify the report as a protocol of a systematic review |  |  | 1 |
| Update | 1b | If the protocol is for an update of a previous systematic review, identify as such |  |  |  |
| **Registration** | 2 | If registered, provide the name of the registry (e.g., PROSPERO) and registration number in the Abstract |  |  |  |
| **Authors** | | | | | |
| Contact | 3a | Provide name, institutional affiliation, and e-mail address of all protocol authors; provide physical mailing address of corresponding author |  |  | 2-15 |
| Contributions | 3b | Describe contributions of protocol authors and identify the guarantor of the review |  |  | 475-480 |
| **Amendments** | 4 | If the protocol represents an amendment of a previously completed or published protocol, identify as such and list changes; otherwise, state plan for documenting important protocol amendments |  |  |  |
| **Support** | | | | | |
| Sources | 5a | Indicate sources of financial or other support for the review |  |  |  |
| Sponsor | 5b | Provide name for the review funder and/or sponsor |  |  |  |
| Role of sponsor/funder | 5c | Describe roles of funder(s), sponsor(s), and/or institution(s), if any, in developing the protocol |  |  |  |
| **INTRODUCTION** | | | | | |
| **Rationale** | 6 | Describe the rationale for the review in the context of what is already known |  |  | 78-143 |
| **Objectives** | 7 | Provide an explicit statement of the question(s) the review will address with reference to participants, interventions, comparators, and outcomes (PICO) |  |  | 162-190 |
| **METHODS** | | | | | |
| **Eligibility criteria** | 8 | Specify the study characteristics (e.g., PICO, study design, setting, time frame) and report characteristics (e.g., years considered, language, publication status) to be used as criteria for eligibility for the review |  |  | 312-332 |
| **Information sources** | 9 | Describe all intended information sources (e.g., electronic databases, contact with study authors, trial registers, or other grey literature sources) with planned dates of coverage |  |  | 196-211 |
| **Search strategy** | 10 | Present draft of search strategy to be used for at least one electronic database, including planned limits, such that it could be repeated |  |  | 196-310 |
| ***STUDY RECORDS*** | | | | | |
| Data management | 11a | Describe the mechanism(s) that will be used to manage records and data throughout the review |  |  | 331-340 |
| Selection process | 11b | State the process that will be used for selecting studies (e.g., two independent reviewers) through each phase of the review (i.e., screening, eligibility, and inclusion in meta-analysis) |  |  | 312-330 |
| Data collection process | 11c | Describe planned method of extracting data from reports (e.g., piloting forms, done independently, in duplicate), any processes for obtaining and confirming data from investigators |  |  | 360-366 |
| **Data items** | 12 | List and define all variables for which data will be sought (e.g., PICO items, funding sources), any pre-planned data assumptions and simplifications |  |  | 173-183 |
| **Outcomes and prioritization** | 13 | List and define all outcomes for which data will be sought, including prioritization of main and additional outcomes, with rationale |  |  |  |
| **Risk of bias in individual studies** | 14 | Describe anticipated methods for assessing risk of bias of individual studies, including whether this will be done at the outcome or study level, or both; state how this information will be used in data synthesis |  |  | 341-358 |
| ***DATA*** | | | | | |
| **Synthesis** | 15a | Describe criteria under which study data will be quantitatively synthesized |  |  |  |
|  | 15b | If data are appropriate for quantitative synthesis, describe planned summary measures, methods of handling data, and methods of combining data from studies, including any planned exploration of consistency (e.g., *I* ^2^, Kendall’s tau) |  |  |  |
|  | 15c | Describe any proposed additional analyses (e.g., sensitivity or subgroup analyses, meta-regression) |  |  |  |
|  | 15d | If quantitative synthesis is not appropriate, describe the type of summary planned |  |  |  |
| **Meta-bias(es)** | 16 | Specify any planned assessment of meta-bias(es) (e.g., publication bias across studies, selective reporting within studies) |  |  |  |
| **Confidence in cumulative evidence** | 17 | Describe how the strength of the body of evidence will be assessed (e.g., GRADE) |  |  |  |
